# Visible-light-promoted decarboxylative radical cascade cyclization to acylated benzimidazo/indolo[2,1-*a*]isoquinolin-6(5*H*)-ones in water†

**DOI:** 10.1039/d2ra03467k

**Published:** 2022-07-07

**Authors:** Lili Tang, Yuejun Ouyang, Kai Sun, Bing Yu

**Affiliations:** Hunan Engineering Research Center for Recycled Aluminum, College of Chemistry & Materials Engineering, Huaihua University Huaihua 418008 China oyyj0816@163.com sunkchem@163.com; College of Chemistry, Zhengzhou University Zhengzhou 450001 China bingyu@zzu.edu.cn

## Abstract

A metal-free visible-light-induced decarboxylative radical addition/cyclization procedure at room temperature was described for the synthesis of acylated benzimidazo/indolo[2,1-a]isoquinolines. The procedure was prepared in water *via* a reaction of functionalized 2-arylbenzoimidazoles or 2,3-diarylindoles and α-oxocarboxylic acids in the presence of phenyliodine(iii) diacetate (PIDA) in one step under mild reaction conditions. In this procedure, traditional heating and metal reagents could be effectively avoided to access 1,4-dicarbonyl-containing benzimidazo/indolo[2,1-*a*]isoquinoline-6(5*H*)-ones in satisfactory yields.

## Abstract 2

The procedure was prepared in water *via* a reaction of functionalized 2-arylbenzoimidazoles or 2,3-diarylindoles and α-oxocarboxylic acids, effectively avoiding traditional heating and metal reagents.

## Graphical abstract

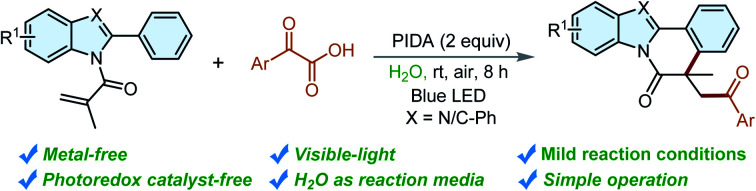

In recent years, photocatalysis has proven to be a sustainable and green synthetic platform which can efficiently convert light energy into chemical energy to form new chemical bonds.^1^ However, most photocatalytic organic conversions are performed in toxic and volatile organic solvents.^2^ Compared with traditional organic solvents, water has unique features to be a promising alternative to volatile solvents with the advantages of being a safe, non-toxic, abundant, and eco-friendly natural source.^3^ Undoubtedly, the development of novel visible-light promoted synthetic methods in water is more in line with the principles of green chemistry, which is highly desired.

Nitrogen-containing heterocyclic compounds play extremely important roles in medicinal chemistry, functional materials, and natural products.^4^ Especially, benzimidazole-isoquinoline fused framework A and indole[2,1-a]isoquinoline containing tetracyclic core structure B are not only widely present in some biologically active molecules, but also as important components of synthetic intermediates and functional materials.^5^ Several representative polyheterocycles containing skeletons A and B with promising applications in the field of treating hemoglobinopathies and cancer, peripheral benzodiazepine receptor ligand, modulating the potassium ion flux or organic electronics are shown in Scheme 1.^6^ Benefit from their significant importance, the developments of novel synthetic method for benzimidazo/indolo[2,1-a]isoquinolin-6(5*H*)-ones have attracted considerable attention in the organic synthetic field.

**Scheme 1:**
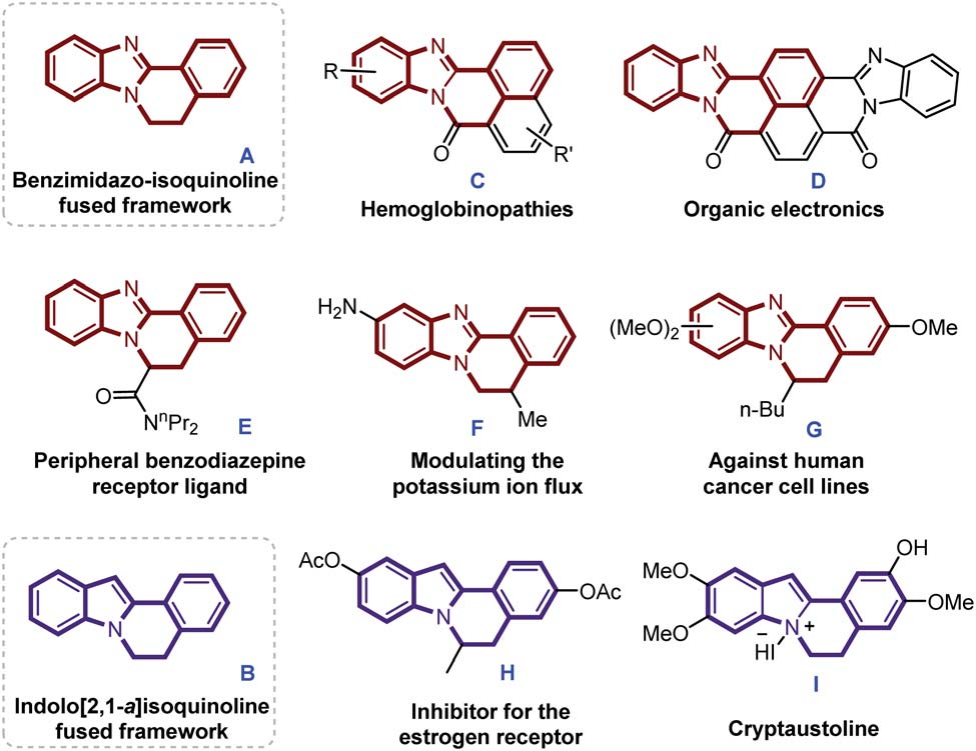
Selected examples of natural products and biologically active molecules.

Recently, α-oxocarboxylic acids have gained much prominence to be acylation reagents owing to their easy preparation, stability, and high reactivity.^7^ However, most transformations that employed α-oxocarboxylic acids as the acyl radical precursor require high-cost noble-metal-based catalysts^8^ or some metal-free organocatalysts.^9^ Radical-initiated cyclizations reaction has developed to be efficient processes for the construction of benzimidazo/indolo-isoquinoline-6(5*H*)-ones,^10^ and the introduction of an acyl group to these meaningful frameworks has been realized using α-oxocarboxylic acids as acyl radical precursors. For instance, our group reported a decarboxylative cascade cyclization of *N*-methacryloyl-2-phenylbenzoimidazole with α-oxocarboxylic acids for the construction of acylated benzimidazo[2,1-*a*]isoquinolin-6(5*H*)-ones in the presence of AgNO_3_/K_2_S_2_O_8_ in 2019 (Scheme 2a).^11^ However, the requirement of a high-cost metal catalyst, high reaction temperature and strong oxidant is unavoidable. Very recently, our group disclosed a metal-free visible-light-induced decarboxylative arylation procedure for the construction of acylated benzimidazo/indolo[2,1-*a*]isoquinolines by using 2,4,5,6-tetra(9H-carbazol-9-yl)isophthalonitrile (4CzIPN) as a photocatalyst in organic solvents (Scheme 2b).^12^ However, the increased manufacturing cost and inconvenient operation of eliminating the photocatalyst impede the applications of these methods in practical application, especially in the pharmaceutical industry. It's still highly desirable to explore a photocatalyst-free approach for the synthesis of acylated benzimidazo/indolo[2,1-*a*]isoquinolines in a sustainable reaction media.

**Scheme 2:**
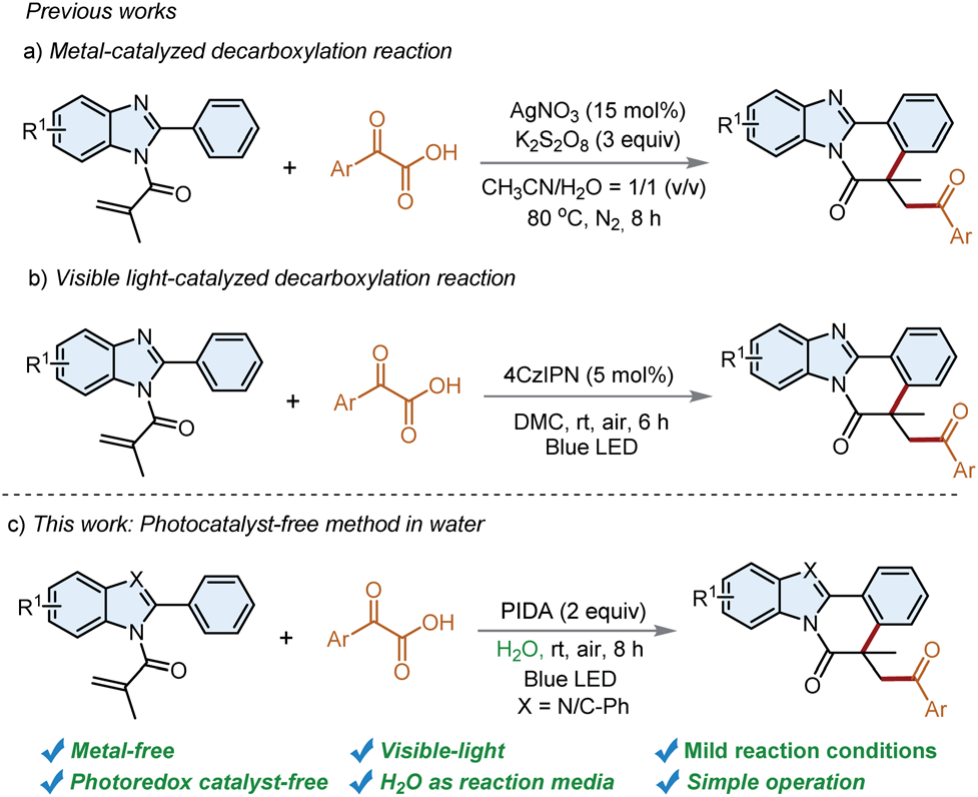
Synthesis of acylated indolo/benzimidazo[2,1-*a*]isoquinolin-6(5*H*)-ones.

With our continuing interests in green synthesis,^13^ we herein describe an environmentally-friendly strategy to access benzimidazo/indolo[2,1-a]isoquinolin-6(5*H*)-ones under the irradiation of blue light. To the best of our knowledge, this is the first example of the synthesis of acylated indolo/benzimidazo[2,1-*a*]isoquinolin-6(5*H*)-ones under irradiation of visible light in water, in which additional photocatalyst and volatile organic solvents could be effectively avoided.

For our initial explorations, we started our study by using 2-methyl-1-(2-phenyl-1H-benzo[*d*]imidazol-1-yl)prop-2-en-1-one (1a) and 2-oxo-2-phenylacetic acid (2a) as the model substrates for the optimization of the reaction conditions in various solvents under irradiation of 5 W blue LED (Table 1). To our delight, this transformation reacted smoothly in presence of PIDA in MeCN or DCE for 8 h to access the desired product in 90% and 61% yield, respectively (entries 1–2). To reduce the impact of solvents on the environment, some green solvents including DMC, PC, H_2_O, 2-CH_3_-THF, EG, TBME, PEG-200, and PEG-400 (entries 2–10) were investigated. Most of the tested solvents could serve as reaction medium, and 80% yield of desired product 3a could be obtained when the reaction was performed in water. Considering that water has the advantages of excellent biocompatibility, non-combustibility and high specific heat capacity, the employment of water as solvents for organic transformation is desirable and attractive.^14^ Therefore, we finally chose H_2_O as the best reaction solvent. To further improve the reaction efficiency, mixed solvents, such as MeOH/water, EtOH/water, THF/water in different ratios were examined (entries 11–16). However, no positive results could be obtained. Next, several control experiments were conducted, which showed that PIDA and light were essential for the transformation (entries 17 and 18). Moreover, the reaction that happened under the N_2_ atmosphere provided slightly lower yields than that in the air (entry 19). Ultimately, the optimal reaction conditions were established as follows: 1a (0.2 mmol), 2a (0.4 mmol), and PIDA (0.4 mmol) in H_2_O at room temperature for 8 h under air atmosphere with the irradiation of 5 W blue LED.

**Table 1:** Optimization of the reaction conditions^a^

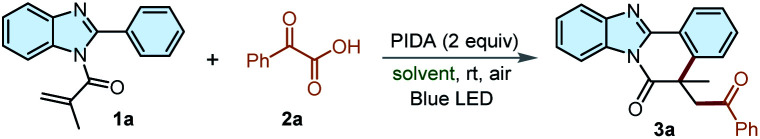		
Entry	Solvent	Yield (%)
1	MeCN	90
2	DCE	61
3	DMC	86
4	PC	Trace
5	H_2_O	80
6	2-CH_3_-THF	42
7	EG	19
8	TBME	57
9	PEG-200	Trace
10	PEG-400	Trace
11	H_2_O : EtOH (*v*/*v* = 1 : 1)	77
12	H_2_O : EtOH (*v*/*v* = 2 : 1)	70
13	H_2_O : MeOH (*v*/*v* = 1 : 1)	78
14	H_2_O : MeOH (*v*/*v* = 2 : 1)	72
15	H_2_O : THF (*v*/*v* = 1 : 1)	61
16	H_2_O : THF (*v*/*v* = 2 : 1)	68
17^b^	H_2_O	N.D.
18^c^	H_2_O	N.D.
19^d^	H_2_O	74

a Reaction conditions: 1a (0.2 mmol), 2a (0.4 mmol), PIDA (0.4 mmol), solvent (2 mL) under air for 8 h, rt, 430 nm Blue LED (5 W). N.D. = Not detected. Isolated yields were given. DCE = 1,2-dichloroethane, DMC = dimethyl carbonate. TBME = *tert*-butyl methyl ether, EG = ethylene glycol, PC = propylene carbonate.

b Without PIDA.

c Experiment performed in the dark.

d Reaction under N_2_ atmosphere.

With the above-optimized reaction conditions established, we subsequently started to explore the substrate scope and limitations of this transformation (Table 2). Initially, a series of α-oxocarboxylic acids 2 were employed as the acyl radical precursors to react with 2-methyl-1-(2-phenyl-1H-benzo[*d*]imidazol-1-yl)prop-2-en-1-one (1a) under the standard reaction conditions. It was pleasing to see that the α-keto acids with both electron-donating and electron-withdrawing substituents were all suitable substrates to be converted into the desired annulation products 3a–3g in good yields (58–80%). Then, *N*-phenylacryloyl-2-phenylbenzoimidazole (1h) was utilized to react with 2a under the standard conditions. The target product 3h was obtained in an 83% yield, demonstrating negligible steric hindrance effects. Afterward, the reactivity of the 2-aryl indoles was evaluated. To our delight, indole derivatives bearing –Me, –F, –Cl, –Br, –CF_3_, and –CN on the indole ring were all compatible to deliver the annulation reaction products 3i–3q in moderate to good yields (43–86%). Notably, an unexpected product 3r was observed when 3,3-dimethyl-2-oxobutyric acid was used as the acyl radical precursor. We assumed that the *in situ* generated 3,3-dimethyl-2-oxobutyric radical could be converted into the more stable *tert*-butyl radical in this process. Regrettably, 1-(2-phenyl-1H-benzo[*d*]imidazol-1-yl)prop-2-en-1-one, 2-methyl-1-(2-phenyl-1H-indol-1-yl)prop-2-en-1-one and 2-thiopheneglyoxylic acid were not suitable substrates in this transformation (3s–u).

**Table 2:** Substrate scope for the synthesis of acylated indolo/benzimidazo[2,1-*a*]isoquinolin-6(5*H*)-ones^a^

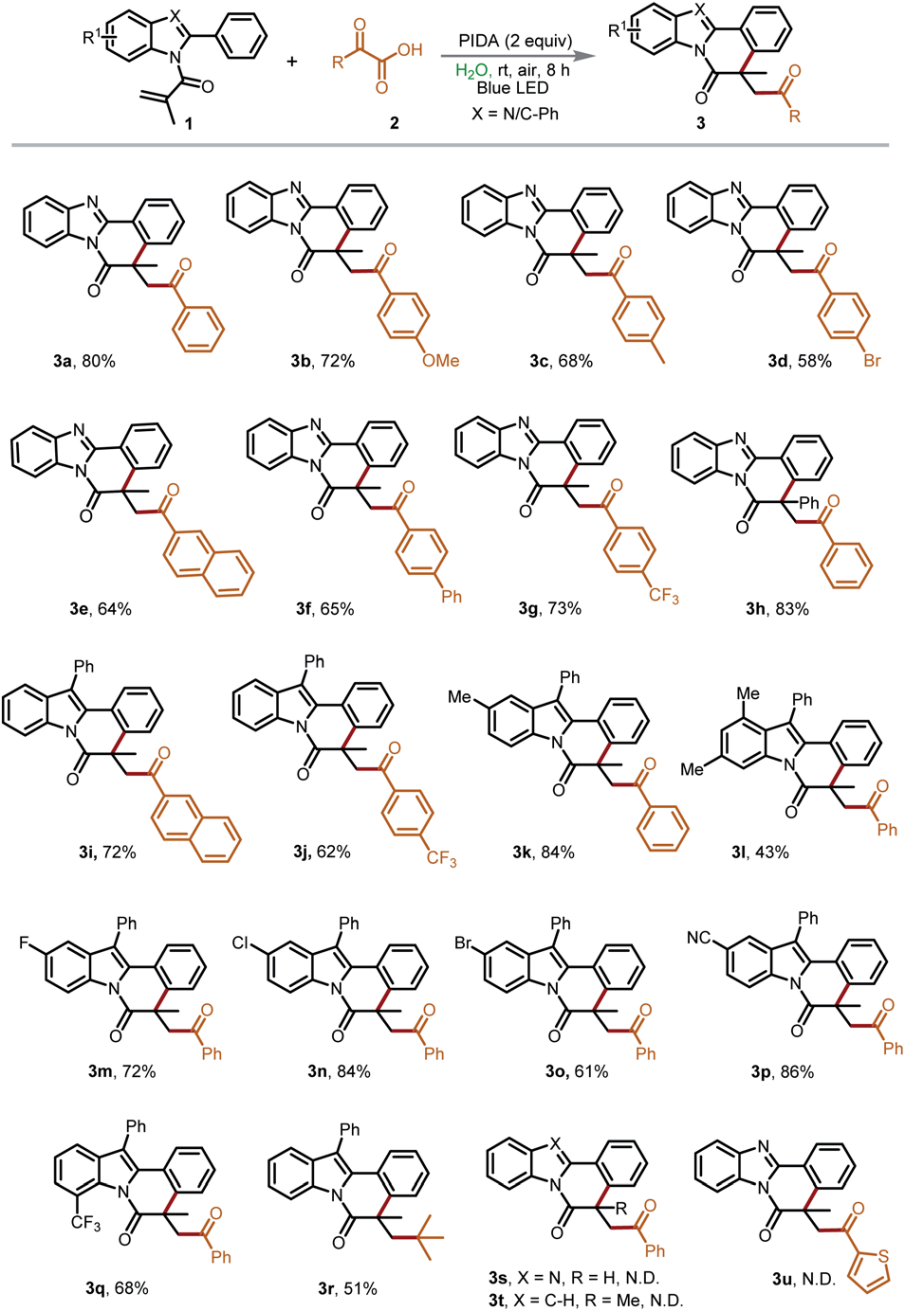

a Reaction conditions: 1 (0.2 mmol), 2 (0.4 mmol) and PIDA (0.4 mmol) in H_2_O (2 mL) with the irradiation of 5 W blue LED under air at room temperature for 8 h. Isolated yields.

Subsequently, we conducted some control experiments to explore the mechanism process in depth. When 3.0 equiv. of 2,2,6,6-tetramethylpiperidin-1-yl-oxidanyl (TEMPO) or 2,6-di-*tert*-butyl-4-methylphenol (BHT) as radical scavengers were subjected to the reaction, the reaction could be completely suppressed, respectively (Scheme 3). Additionally, the complex of TEMPO-benzoyl radical was observed by high-resolution mass spectrometry (HRMS), indicating that a radical process might be involved in this transformation (Fig. S2†).

**Scheme 3:**
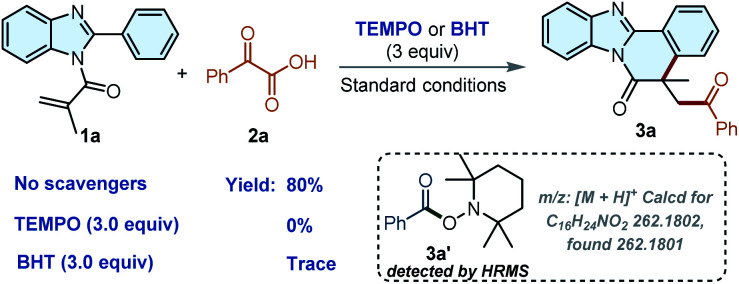
Control Experiments.

Based on the experimental results and previous reports,^15^ a plausible mechanism for the reaction was thus proposed as illustrated in Scheme 4. First, a ligand exchange interaction between PIDA and 2a led to the formation of hypervalent iodine(iii) reagent A, which underwent I–O bond cleavage under the irradiation of visible light to deliver radicals B and C. Subsequently, the benzoyl radical could be generated *via* the fragmentation of B and C. The benzoyl radical was then added to the C=C bond of 1a to afford a radical species D, which underwent an intramolecular cyclization to give radical E. Radical E was then converted into carbocation F by PIDA or oxygen through a single-electron oxidation process. Finally, product 3a could be formed by rapid deprotonation of the carbon cation F.

**Scheme 4:**
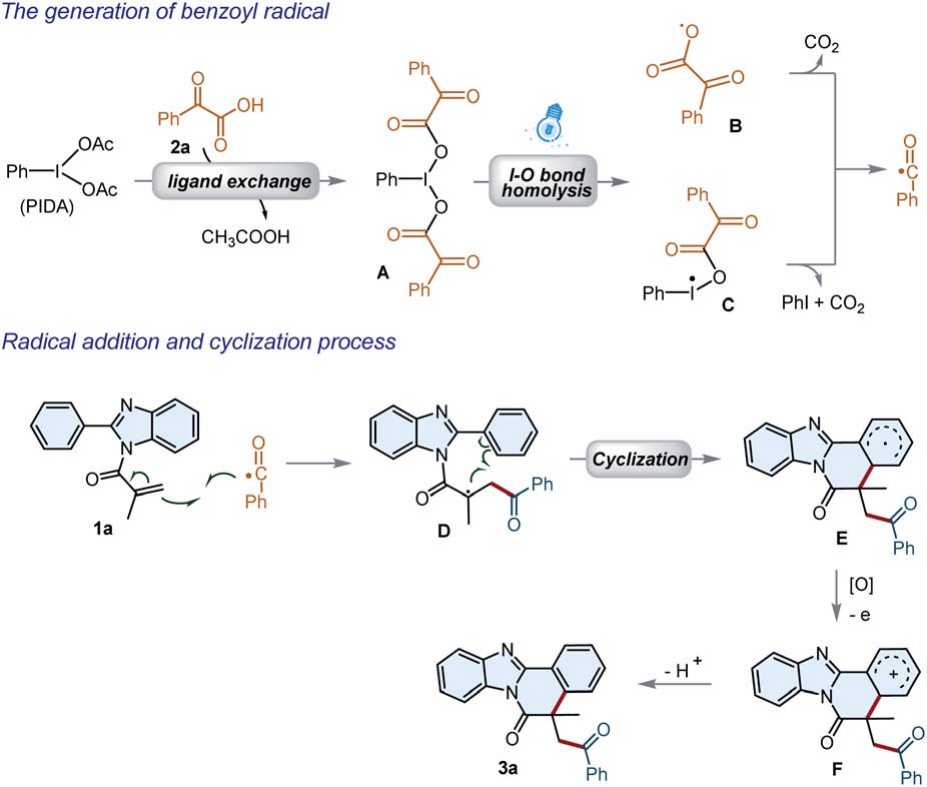
Proposed Reaction Mechanisms.

## Conclusions

In summary, we have successfully disclosed a metal-free visible-light-induced decarboxylative radical cyclization process to construct acylated benzimidazo/indolo[2,1-*a*]isoquinolines in water under photoredox catalyst-free conditions. The remarkable advantages of this strategy include photoredox catalyst and metal-free reaction conditions, sustainable reaction medium, room reaction temperature, and high efficiency. Further explorations on the applications of visible-light-induced organic synthetic transformations are currently ongoing in our laboratory.

## Conflicts of interest

There are no conflicts to declare.

## Supplementary Material
